# A Data Compression Method for Mechanical Vibration Wireless Sensor Networks Based on Data Segmentation and Non-Uniform Quantization

**DOI:** 10.3390/e28101156

**Published:** 2026-10-20

**Authors:** Yi Huang, Jiaquan Zhang, Xiaojing Mu, Dongmei Tian, Rongtao Mu, Tianhong Luo

**Affiliations:** 1 School of Intelligent Manufacturing, Chongqing University of Arts and Sciences, Chongqing 402160, China; 2 Key Laboratory of Optoelectronic Technology & Systems Ministry of Education, International R & D Center of Micro-Nano Systems and New Materials Technology, Chongqing University, Chongqing 400044, China; 3 Department of Information and Intelligence Engineering, Chongqing City Vocational College, Chongqing 402160, China

**Keywords:** mechanical vibration monitoring, wireless sensor networks, data compression, data segmentation, non-uniform quantization, variable run-length encoding, range coding

## Abstract

High-frequency sampling in mechanical vibration wireless sensor networks (WSNs) generates large volumes of raw data, imposing substantial demands on transmission bandwidth and node energy consumption. To address this problem, this paper proposes a two-stage compression method based on data segmentation and non-uniform quantization. First, byte-level statistical characteristics of 24-bit raw analog-to-digital converter (ADC) data are analyzed. The results reveal the limitations of conventional transform-domain thresholding and show distinct statistical characteristics among different bit planes. Accordingly, each 24-bit sample is divided into an 8-bit sign-bit channel and a 16-bit content-bit channel. The sign-bit channel exhibits high temporal redundancy and is therefore compressed losslessly using variable run-length encoding (VRLE). An adaptive repetition-ratio criterion is introduced to select the encoding mode and avoid data expansion for short repetition sequences. The content-bit channel is compressed using an eight-interval non-uniform quantization scheme inspired by the A-Law PCM standard. Finer quantization is assigned to small-amplitude components to reduce reconstruction errors. Range coding is subsequently applied to both encoded streams to further remove statistical redundancy. Experiments on periodic rotor vibration signals achieve a compression ratio of 3.22 and a reconstruction signal-to-noise ratio (RSNR) of 37.4 dB. For aperiodic modal testing signals, compression ratios of 3.16 and 2.66 are obtained for force-hammer excitation and frame response signals, with RSNR values of 46.49 and 54.2 dB, respectively. Furthermore, modal identification using the reconstructed data results in a maximum relative error of only 0.03% for the first five natural frequencies. These results demonstrate that the proposed method effectively reduces vibration data volume while preserving critical dynamic characteristics.

## 1. Introduction

Mechanical vibration monitoring is widely used for condition assessment and fault diagnosis of mechanical equipment [1,2,3]. Wireless sensor networks (WSNs) provide flexible deployment, distributed sensing, and low-cost data acquisition, making them attractive for large-scale vibration monitoring applications. However, high-fidelity vibration measurements generally require high-resolution analog-to-digital converters (ADCs) and high sampling rates. ADC resolutions of 16–24 bits and sampling frequencies above 10 kHz can generate large volumes of raw data at each sensor node. This places substantial demands on wireless transmission bandwidth and node energy consumption, particularly in resource-constrained WSNs. Therefore, reducing the amount of vibration data before wireless transmission is essential for efficient and reliable mechanical vibration monitoring [4,5].

Considerable research has been devoted to wireless sensing for vibration-based monitoring. Existing studies have addressed fundamental aspects of WSNs, including network architectures, communication protocols, sensing technologies, data acquisition, and energy management. Wireless sensing systems have also been investigated for distributed vibration measurement, structural dynamic parameter identification, and vibration-based structural health monitoring [6,7,8]. Energy-aware network configurations have further improved the efficiency of multi-node sensing and data transmission [9]. These advances have demonstrated the feasibility of WSNs for distributed vibration monitoring. Nevertheless, the increasing resolution and sampling frequency of vibration acquisition lead to a rapid growth in the amount of raw data generated at sensor nodes [10]. In contrast, WSNs generally provide limited communication bandwidth. For example, IEEE 802.15.4 offers a nominal physical-layer data rate of only 250 kbps, while the actual application-layer throughput can be further reduced by protocol overhead, channel contention, backoff procedures, and multi-hop transmission. Consequently, the mismatch between the high data generation rate of vibration sensing and the limited transmission capacity of WSNs has become a major bottleneck in high-fidelity wireless vibration monitoring.

To alleviate this data-transmission burden, various compression and data-reduction techniques have been investigated for WSNs. Existing approaches include dictionary-based, prediction-based, and transform-domain compression, as well as adaptive sampling and distributed source coding [11,12]. Compressed sensing (CS) provides another framework for reducing the amount of data required for signal representation by exploiting signal sparsity [13,14]. Transform-domain methods based on the discrete cosine transform (DCT) and wavelet transform have also been widely adopted because of their energy compaction and time–frequency localization capabilities [15,16]. In addition, CS has been integrated with network-level strategies such as clustering to improve the energy efficiency of wireless data transmission [17]. These methods have demonstrated considerable potential for reducing communication and storage requirements in WSNs.

Despite these advances, existing compression methods present several limitations when applied to high-resolution raw mechanical vibration data on resource-constrained sensor nodes. Transform-domain and CS-based approaches often require additional transformations, matrix operations, or threshold selection, which increase computational and memory requirements. More importantly, most existing methods process vibration samples or transformed coefficients, without explicitly exploiting the different statistical characteristics associated with the byte-level structure of high-resolution ADC data. For 24-bit mechanical vibration samples, different bit segments may exhibit substantially different levels of temporal redundancy and amplitude information. Applying a uniform compression strategy to all bits may therefore fail to fully exploit this structural redundancy. This motivates a compression strategy that operates directly on raw ADC data and applies differentiated processing according to the statistical characteristics of different data segments.

To address these limitations, this study proposes a two-stage compression method based on data segmentation and non-uniform quantization for high-resolution mechanical vibration WSNs. The method first analyzes the byte-level statistical characteristics of 24-bit raw ADC data and divides each sample into an 8-bit sign-bit segment and a 16-bit content-bit segment. The sign-bit segment exhibits strong temporal redundancy and is compressed losslessly using variable run-length encoding (VRLE). In contrast, the content-bit segment carries the principal amplitude information and is compressed using non-uniform quantization to control reconstruction distortion. Range coding is subsequently applied to both encoded streams to further eliminate statistical redundancy. The proposed method is designed to reduce data volume while maintaining the dynamic characteristics required for mechanical vibration analysis.

The key contributions of this paper are summarized as follows:

1. A byte-level data segmentation strategy is proposed for 24-bit raw mechanical vibration ADC data. The strategy separates the sign-bit and content-bit segments according to their distinct statistical characteristics, providing a basis for differentiated compression.
2. A VRLE scheme with an adaptive repetition-ratio criterion is developed for the sign-bit segment. The scheme adaptively selects the encoding mode to achieve lossless compression while avoiding data expansion for short repetition sequences.
3. A non-uniform quantization scheme is developed for the 16-bit content-bit segment. The numerical space is divided into eight non-uniform intervals to provide finer resolution for small-amplitude components. Range coding is further applied to reduce residual statistical redundancy.
4. The proposed method is implemented on a self-developed dual-core WSN node and evaluated using both periodic rotor vibration and aperiodic modal testing signals. The experimental results demonstrate a compression ratio of approximately 3:1 while preserving the dynamic characteristics required for vibration and modal analysis.

The remainder of this paper is organized as follows. Section 2 reviews related compression methods and analyzes the byte-level characteristics of raw mechanical vibration ADC data. Section 3 presents the proposed two-stage compression method, including block-based processing, VRLE, non-uniform quantization, and Range coding. Section 4 describes the experimental platform and evaluates the proposed method using periodic and aperiodic vibration signals. Finally, Section 5 concludes the paper and discusses future work.

## 2. Related Work and Byte-Level Characteristics of Raw ADC Data

### 2.1. Related Works

Data compression methods for WSNs can generally be divided into several categories according to their underlying principles. Lossless approaches, such as dictionary-based and prediction-based methods, preserve the original data exactly and are suitable for applications requiring complete reconstruction. However, their compression performance is often limited when applied to high-dynamic-range vibration signals with weak temporal predictability. Hybrid lossless/lossy strategies have therefore been investigated to achieve a better trade-off between compression efficiency and computational cost [11,18].

Transform-domain compression represents another widely used category. Typical approaches employ the discrete cosine transform (DCT) or wavelet transform to concentrate signal energy into a limited number of coefficients, after which insignificant coefficients are discarded or quantized [15,16,19]. These methods are effective when the signal energy is highly concentrated in the transform domain. However, their performance depends strongly on coefficient distributions and threshold selection. For non-stationary or broadband mechanical vibration signals, informative components may also appear outside the dominant low-frequency region, making fixed thresholding potentially unreliable.

CS provides an alternative framework by exploiting signal sparsity and reconstructing signals from a reduced number of measurements [13,14,20]. It has also been combined with clustering and network-level transmission strategies to reduce communication energy in WSNs [17]. Although CS can achieve substantial data reduction, it usually requires matrix operations and iterative reconstruction, which may increase computational complexity at resource-constrained nodes or data centers. Recent deep-learning-based approaches have also been applied to signal compression and reconstruction for industrial condition monitoring [21].

In addition to compression algorithms, other data-reduction strategies, including adaptive sampling, in-network aggregation, and distributed source coding, have also been studied for WSN applications [12]. Compression and reconstruction methods have also been combined with fault diagnosis for vibration signals [22], while deep-learning-based fault diagnosis has attracted increasing attention [23]. These methods reduce communication load from the perspectives of sensing, networking, or collaborative processing. Nevertheless, most existing approaches process vibration data as complete samples or transform-domain coefficients. The internal byte-level structure of high-resolution raw ADC data has received relatively limited attention. This motivates the investigation of compression methods that directly exploit the statistical differences among different byte segments of raw mechanical vibration data.

### 2.2. Transform-Domain Analysis of Raw Vibration ADC Data

Transform-domain methods achieve data reduction by concentrating signal energy into a limited number of coefficients and discarding less significant components. Their compression performance therefore depends strongly on the distribution of transform coefficients and the criterion used for coefficient retention. To evaluate the applicability of this strategy to high-resolution mechanical vibration data, the transform-domain characteristics of raw ADC samples are analyzed in this section. The relationship between the measured vibration quantity and the corresponding raw ADC output is established based on the sensor sensitivity and the ADC conversion characteristics. Specifically, the ADC input voltage at sampling point *i* can be expressed as:

(1) $$V_{i}=\frac{x_{\mathrm{nbit}}^{i}}{2^{\mathrm{nbit}}}\times \left(2V_{\mathrm{ref}}\right)$$

Since the sensor sensitivity *K* is defined as the ratio between the sensor output voltage and the corresponding measured vibration quantity, the vibration quantity can be obtained as:

(2) $$x_{i}=\frac{V_{i}}{K}=\frac{1}{K}\times \frac{x_{\mathrm{nbit}}^{i}}{2^{\mathrm{nbit}}}\times \left(2V_{\mathrm{ref}}\right)$$

where *x_i_* denotes the measured vibration quantity at sampling point *i*, *K* denotes the sensor sensitivity, *nbit* denotes the ADC resolution in bits, $x_{\mathrm{nbit}}^{i}$ denotes the *n*-bit raw ADC output at sampling point *i*, and *V_ref_* denotes the ADC reference voltage. Equation (2) is therefore a deterministic conversion relationship derived from the sensor sensitivity and ADC quantization characteristics, rather than an empirical relationship obtained from the experimental data.

As shown in Equation (2), the measured vibration signal is linearly related to the raw ADC output through a constant scaling factor. Therefore, the statistical and transform-domain characteristics relevant to compression can be analyzed directly from the raw ADC data. To investigate these characteristics, vibration data from the Case Western Reserve University (CWRU) Bearing Data Center are used [24]. The sensor sensitivity is set to 100 mV/g, with a 24-bit ADC resolution and a reference voltage of 5 V. Previous research has investigated the influence of block size on data compression performance in WSNs [25]. Based on this prior work and the resource constraints of the developed sensor node, a block size of 2048 samples was adopted for the present analysis and the proposed compression framework. The rationale for this selection is further discussed in Section 3.1.

**Figure 1:**
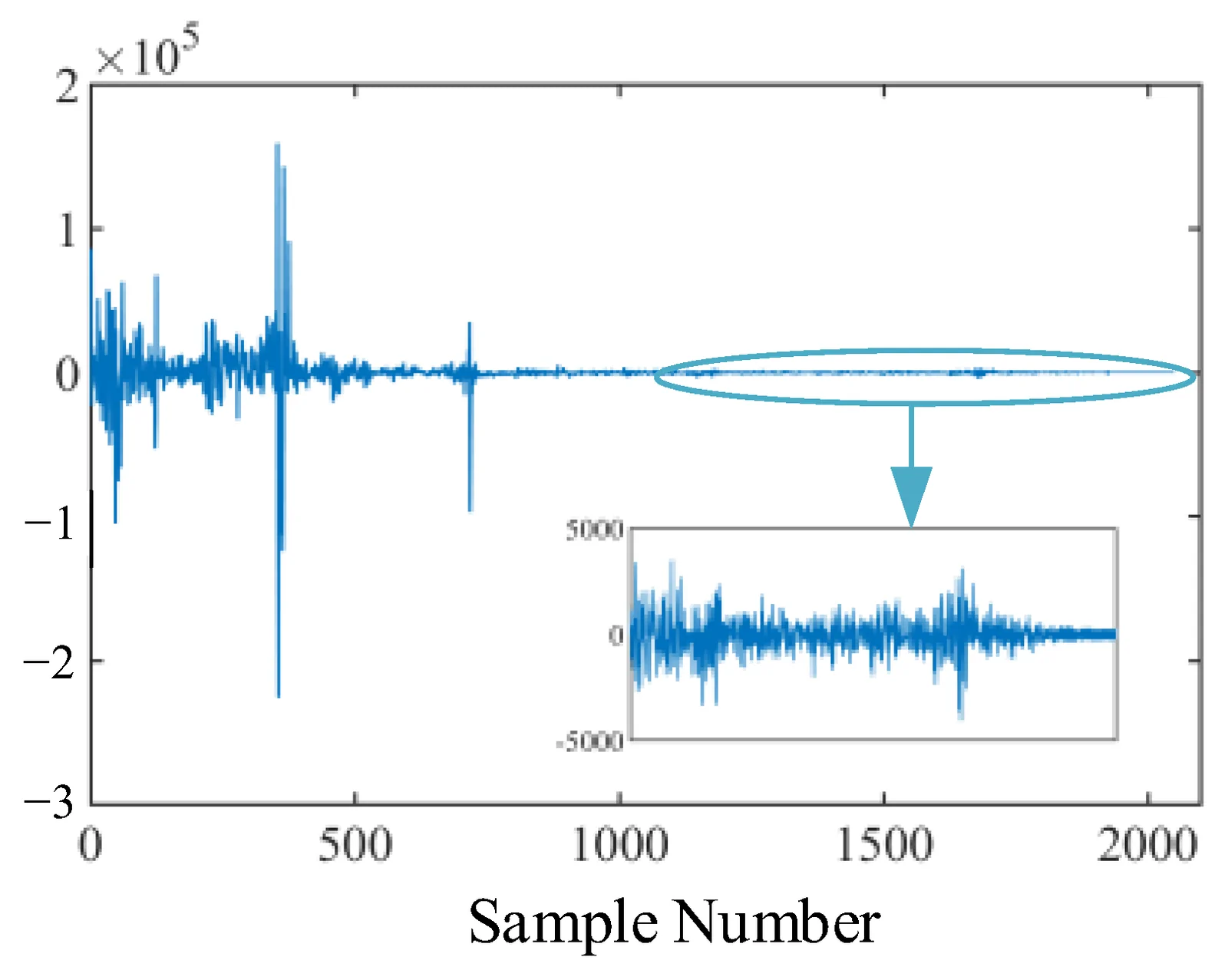
DCT coefficients of raw mechanical vibration ADC data.

The DCT is then applied to each data block to examine the energy distribution of the vibration signal in the transform domain. As shown in Figure 1, the dominant signal energy is concentrated in a relatively small number of low-frequency coefficients. This energy-compaction property provides the basis for reducing data by retaining significant coefficients while discarding less important ones. However, a considerable number of coefficients remain outside the dominant low-frequency region, with amplitudes approximately ranging from −5000 to +5000. These coefficients cannot simply be regarded as redundant information.

Applying a fixed threshold to remove these coefficients may lead to two problems. First, the distribution and magnitude of the transform coefficients vary with operating conditions and signal characteristics. A fixed threshold may therefore be difficult to generalize across different vibration signals. Second, mechanical vibration signals can contain transient, aperiodic, or multi-frequency components. Some coefficients with relatively small amplitudes may still carry useful dynamic information. Excessive coefficient truncation may consequently introduce distortion or remove information relevant to subsequent vibration analysis.

These observations indicate that transform-domain compression involves a trade-off between data reduction and information preservation. Moreover, transform operations and coefficient-selection procedures introduce additional computational requirements for resource-constrained WSN nodes. Therefore, instead of relying solely on transform-domain sparsity, it is necessary to explore the intrinsic redundancy of raw ADC data from another perspective. In the following section, the byte-level statistical characteristics of the raw ADC samples are further analyzed to provide the basis for the proposed data-segmentation strategy.

### 2.3. Byte-Level Characteristics of Raw Mechanical Vibration ADC Data

Motivated by the limitations of transform-domain compression discussed above, the statistical characteristics of the raw ADC data are further investigated at the byte level. A 24-bit ADC sample contains information with different numerical significance across its bit positions. To examine whether these differences can be exploited for compression, each 24-bit sample is represented as three 8-bit fields: the upper byte (Bits 23–16), the middle byte (Bits 15–8), and the lower byte (Bits 7–0). This byte-wise representation also avoids padding each 24-bit sample into a 32-bit data type during storage and processing on embedded platforms.

The three byte fields play different roles in representing the vibration waveform. The upper byte contains the sign information together with the most significant amplitude bits and therefore tends to change relatively slowly between consecutive samples. It is referred to as the sign-bit segment in this study. The middle byte primarily reflects the main amplitude variation in the waveform and is termed the primary content-bit segment. The lower byte represents finer amplitude variations and is termed the detail content-bit segment. The temporal and numerical distributions of these three segments are analyzed below to determine their respective redundancy characteristics.

**Figure 2:**
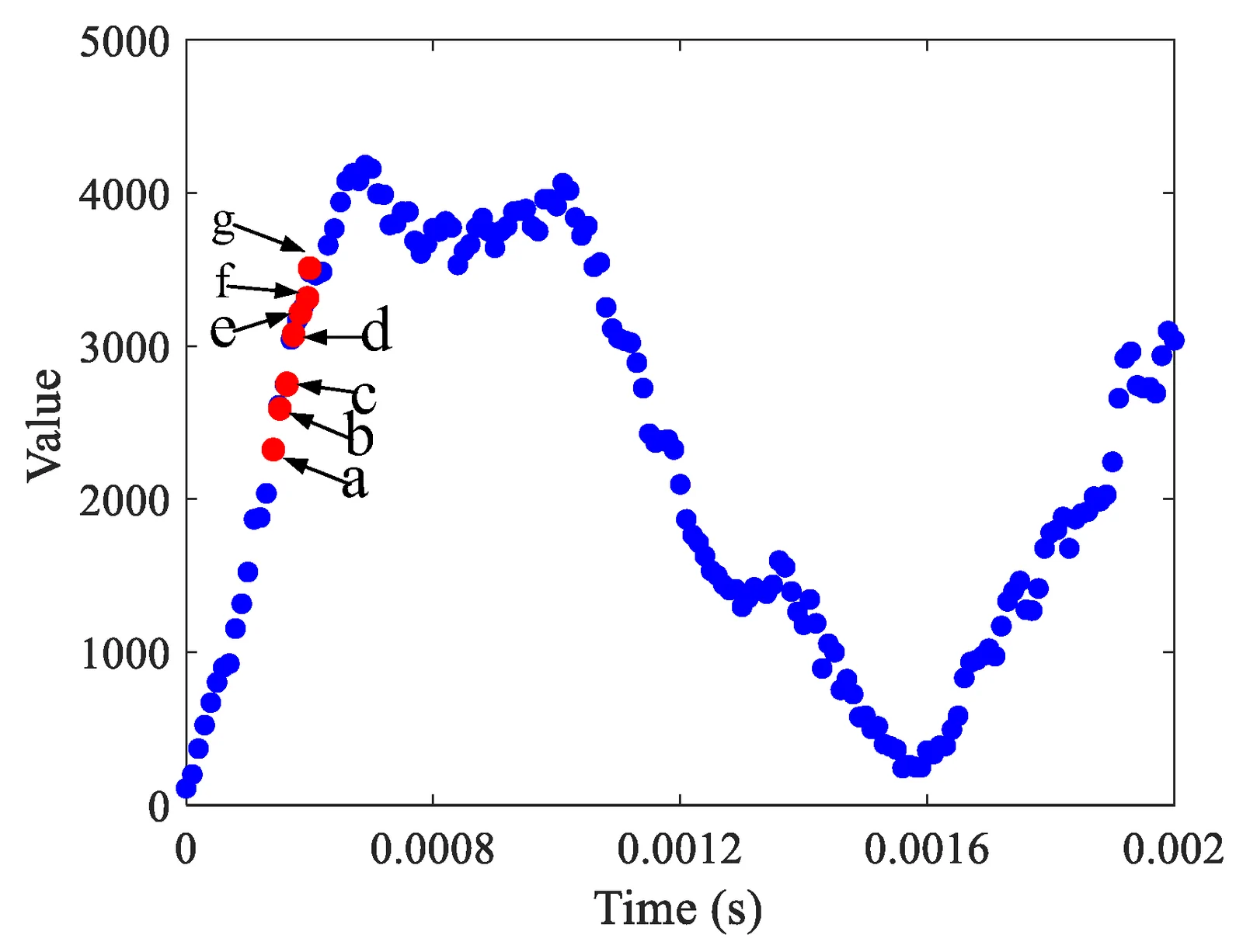
Representative vibration waveform and selected ADC samples.

Figure 2 shows a representative time-domain waveform acquired by the self-developed WSN node. To illustrate the byte-level variation in the raw ADC data, seven consecutive samples, labeled a–g, are selected from the waveform. Their decimal values and corresponding 24-bit two’s-complement representations are listed in Table 1, where each sample is divided into upper, middle, and lower 8-bit fields.

As shown in Table 1, the three-byte fields exhibit distinctly different variation patterns. Although the sample values increase from 2037 to 3256, the upper byte remains unchanged at “00000000” for all seven samples. In contrast, the middle byte changes from “00000111” to “00001100”, while the lower byte varies more frequently between consecutive samples. These observations indicate that the upper byte exhibits stronger temporal redundancy than the middle and lower bytes and therefore contains greater redundancy that can be exploited by lossless compression. The middle and lower bytes, in contrast, carry most of the sample-to-sample amplitude variation and should be retained with appropriate numerical resolution.

To further investigate the byte-level characteristics of raw 24-bit ADC data, a quantitative statistical analysis was conducted using 267,540 vibration samples acquired by the developed WSN node. The number of distinct byte values, repetition ratio, and average run length were calculated separately for the upper, middle, and lower bytes. The repetition ratio was defined as the proportion of adjacent byte values that were identical. The results are summarized in Table 2.

As shown in Table 2, the upper byte contains only two distinct values (0x00 and 0xFF), with a repetition ratio of 98.09% and an average run length of 52.44. In contrast, the middle byte exhibits a repetition ratio of 52.24% and an average run length of 2.09, while the lower byte has a repetition ratio of only 0.40% and an average run length of approximately 1.00. These results indicate that the upper byte exhibits substantially stronger temporal redundancy than the middle and lower bytes. This characteristic can be attributed to the limited amplitude range of the acquired vibration signals relative to the full-scale range of the 24-bit ADC, resulting in repeated sign-extension patterns in the high-order bits. The quantitative results therefore support the proposed differentiated compression strategy.

**Figure 3:**
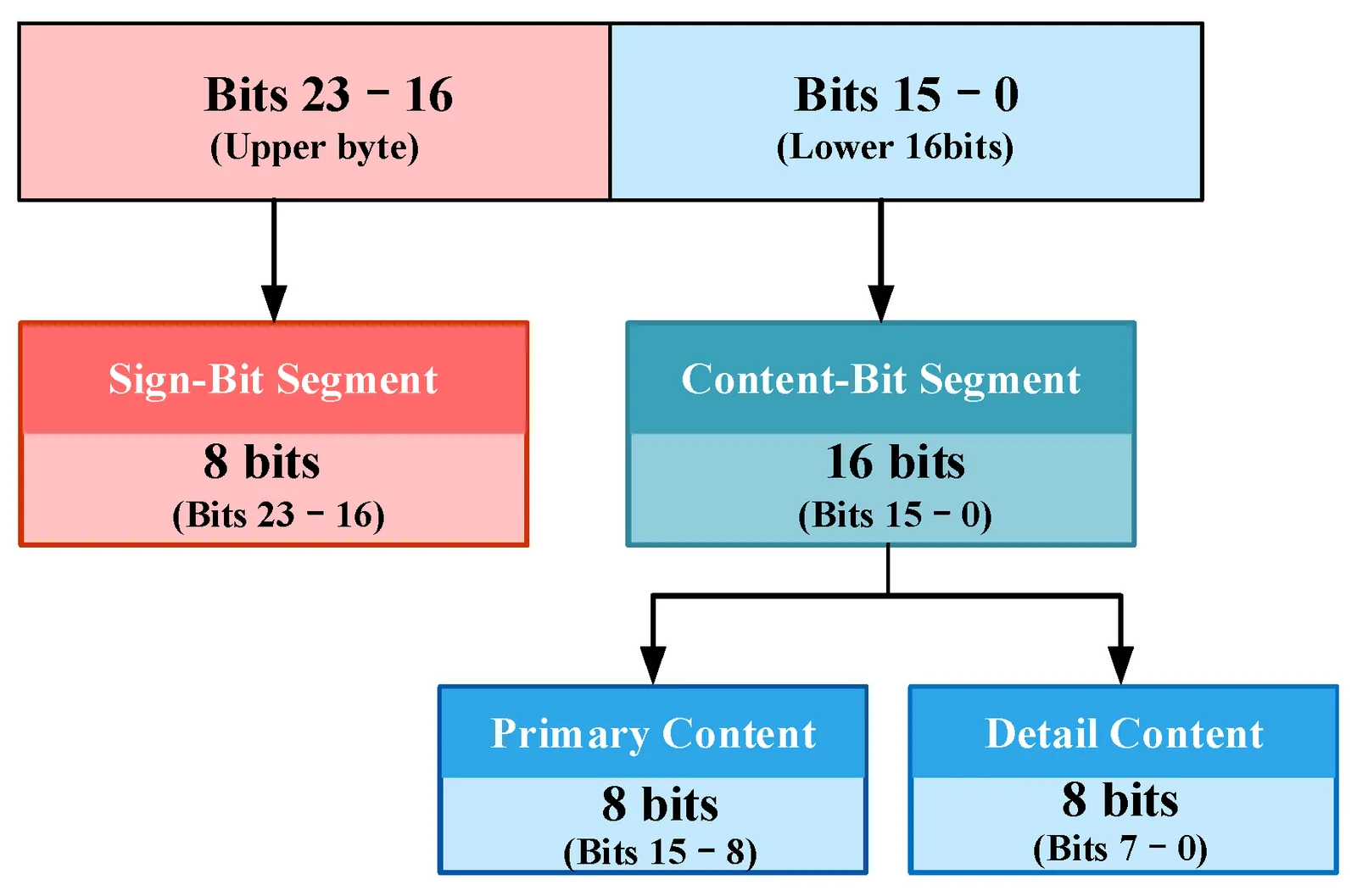
Byte-level segmentation of a 24-bit ADC sample.

Based on the above byte-level characteristics, the 24-bit raw ADC data are reorganized into two segments for differentiated compression. The upper byte (Bits 23–16), which exhibits strong temporal redundancy, is defined as the 8-bit sign-bit segment. The middle and lower bytes (Bits 15–0), which carry most of the sample-to-sample amplitude variation, are combined to form the 16-bit content-bit segment. The two segments are then processed through separate compression pipelines, as illustrated in Figure 3. This segmentation strategy enables the compression method to exploit their distinct statistical characteristics rather than applying a uniform compression scheme to the entire 24-bit sample.

### 2.4. Architecture of the Proposed Data Compression System

**Figure 4:**
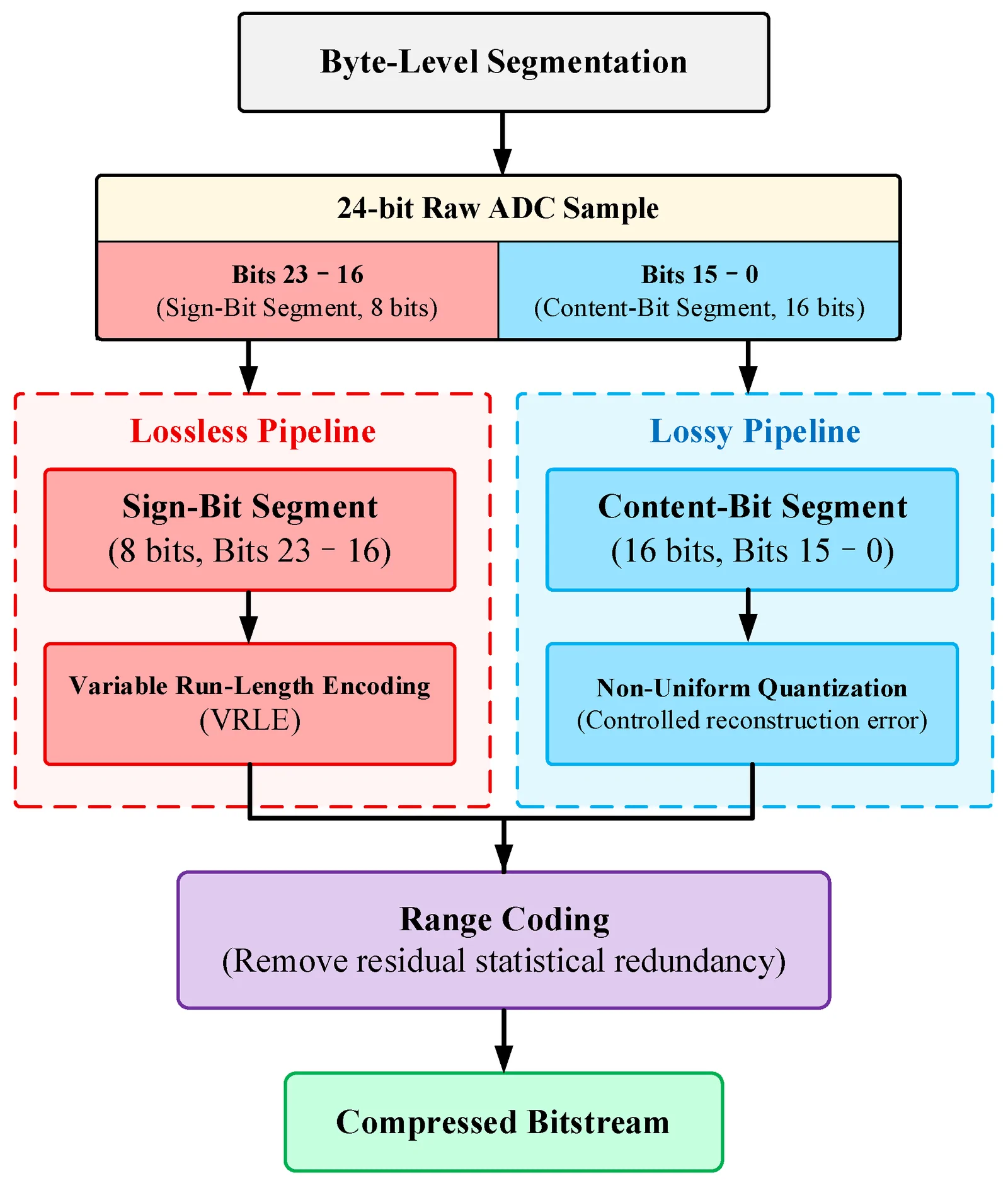
Architecture of the proposed data-segmentation-based non-uniform quantization compression method.

Based on the above data-segmentation strategy, the overall architecture of the proposed compression method is shown in Figure 4. Each 24-bit raw ADC sample is first divided into an 8-bit sign-bit segment and a 16-bit content-bit segment. The two segments are then processed by separate compression pipelines according to their statistical characteristics. The sign-bit segment is compressed losslessly using VRLE, whereas the content-bit segment is compressed using non-uniform quantization with controlled reconstruction distortion. The resulting data streams are subsequently processed by Range coding to further reduce residual statistical redundancy and generate the final compressed bitstream.

The differentiated compression strategy is designed to balance data reduction and signal reconstruction accuracy. Lossless compression is applied to the sign-bit segment because it contains the sign and high-order amplitude information of the raw ADC samples. Errors in this segment may therefore cause substantial distortion in the reconstructed waveform. In contrast, controlled quantization is applied to the content-bit segment to achieve greater data reduction while limiting reconstruction error. Since quantization distortion is closely related to the quantization step size, finer quantization can be assigned to amplitude regions requiring higher numerical resolution [26]. Accordingly, a non-uniform quantization scheme is adopted for the content-bit segment, with finer resolution for small-amplitude values and progressively coarser resolution for larger amplitudes.

The three compression components serve complementary rather than interchangeable functions in the proposed framework. VRLE exploits the temporal redundancy of the sign-bit segment through lossless coding, while non-uniform quantization provides the primary data reduction for the content-bit segment with controlled distortion. Range coding is subsequently applied to the outputs of both branches as a lossless entropy-coding step to remove residual statistical redundancy. Therefore, these components operate on different representations or stages of the data and collectively form the proposed compression pipeline.

The compression workflow is performed on a block-by-block basis. Raw 24-bit ADC samples are first read from the Micro SD card in fixed-size blocks. Each sample is then divided into an 8-bit sign-bit segment and a 16-bit content-bit segment, forming two independent data streams for subsequent compression. For the sign-bit stream, the repetition characteristics of consecutive symbols are analyzed within each block. The repetition ratio *R* is calculated and used to adaptively select the appropriate VRLE mode for lossless compression. For the content-bit stream, eight-interval non-uniform quantization is applied to reduce the data volume with controlled reconstruction distortion. The resulting streams are subsequently encoded using Range coding to further remove statistical redundancy. The compressed outputs are then combined to form the final bitstream for wireless transmission. At the data center, the original data structure is reconstructed by performing the corresponding decoding operations in reverse order.

The detailed implementations of VRLE, non-uniform quantization, and Range coding are presented in Section 3.

## 3. Proposed Two-Stage Compression Method

### 3.1. Block-Based Data Processing

The limited SRAM capacity of embedded microcontrollers restricts the amount of vibration data that can be processed at one time. The STM32F405 used in the sensor node provides 192 KB of SRAM, whereas a single acquisition may generate several hundred kilobytes of raw data. Therefore, the complete dataset cannot be loaded into SRAM for one-pass compression, and a block-based processing strategy is adopted.

In this study, each data block contains 2048 samples, corresponding to 6 KiB of 24-bit raw ADC data. During compression, one block is read from the Micro SD card into SRAM through the SDIO interface and processed independently. After compression is completed, the resulting bitstream is output before the next block is loaded. This sequential processing scheme limits the SRAM occupancy and avoids simultaneous access to the data buffer during SDIO transfer and compression.

The block size affects compression efficiency, memory usage, and processing latency. Previous research has investigated the influence of block size on compression performance in WSNs [25], providing a useful reference for block-size selection. For 24-bit ADC data, each sample occupies 3 bytes, corresponding to raw block sizes of 0.75, 1.5, 3, 6, and 12 KiB for 256, 512, 1024, 2048, and 4096 samples, respectively. Smaller blocks provide a shorter statistical context for VRLE and range coding and may increase the relative overhead associated with block management and coding initialization. Conversely, larger blocks require more buffer space and may increase block-processing latency. Considering the findings of the previous study [25], together with these trade-offs and the resource constraints of the STM32F405-based sensor node, a block size of 2048 samples (6 KiB) was adopted as a practical engineering configuration rather than a theoretically optimal value.

### 3.2. VRLE for the Sign-Bit Segment

As discussed in Section 2.3, the sign-bit segment exhibits strong temporal redundancy, with identical byte values often occurring over consecutive samples. This characteristic makes run-length encoding (RLE) a suitable candidate for lossless compression. RLE represents a sequence of consecutive identical symbols by storing the symbol and its run length [27,28]. For example, the sequence “AAAACCCCCDDD” can be encoded as (A,4), (C,5), and (D,3), from which the original sequence can be exactly reconstructed.

However, conventional RLE does not guarantee a positive compression gain. When a symbol occurs only once, storing both the symbol and its run length introduces additional overhead. Even for short runs, the reduction in data size may be insufficient to compensate for the additional run-length field. Therefore, applying a fixed RLE format to all sign-bit data may result in inefficient encoding or even data expansion when short runs occur frequently. Zero-run-length encoding (Zero-RLE) alleviates this problem by encoding only consecutive zeros. However, repeated values in the sign-bit stream are not restricted to zero. Consequently, Zero-RLE cannot fully exploit the temporal redundancy of the sign-bit segment.

To address these limitations, a VRLE scheme is developed in this study. The proposed scheme evaluates the repetition characteristics within each data block and adaptively selects the encoding mode according to the repetition ratio *R*, thereby retaining the advantages of RLE for long repeated sequences while avoiding unnecessary coding overhead for short or non-repeated sequences.

The proposed VRLE scheme adaptively switches among three encoding modes—full run-length mode, mixed mode, and raw pass-through mode—according to the repetition characteristics of the sign-bit stream within each data block. For a sign-bit block containing *N* symbols, the block is first scanned to identify consecutive runs of identical symbols. Let *n_i_* denote the run length of the *i*-th repeated sequence. The repetition ratio *R* is then defined as:

(3) $$R=\frac{2\times \sum k_{i}^{1}}{N}$$

Based on the calculated repetition ratio *R*, the VRLE encoder adaptively selects one of three encoding modes. Two thresholds, *R_H_* and *R_L_*, are introduced to distinguish blocks with different levels of temporal redundancy, where *R_H_* = 0.6 and *R_L_* = 0.3. Their influence on compression performance is further evaluated through a sensitivity analysis in Section 4.3. The encoding mode is selected according to the following rule:

(1) Full run-length mode (*R* ≥ *R_H_*): All consecutive runs in the sign-bit block are represented by (symbol, run length) pairs. This mode is used for blocks with strong temporal redundancy, where the reduction achieved by run-length encoding outweighs the additional coding overhead.
(2) Mixed mode (*R_L_* ≤ *R* < *R_H_*): Only runs with *n_i_* > 2 are run-length encoded, whereas short runs and isolated symbols are retained in their original form. Mode flags are appended to distinguish run-length-coded units from raw units during decoding. This mode exploits sufficiently long repeated sequences while avoiding unnecessary overhead for short runs.
(3) Raw pass-through mode (*R* < *R_L_*): The sign-bit block is transmitted without run-length encoding. This mode avoids the data expansion that may occur when RLE is applied to blocks with weak temporal redundancy.

An important consideration in the embedded implementation of VRLE is the management of the encoding buffer. Because the encoded data size depends on the run-length distribution and the selected encoding mode, the required buffer size cannot be determined directly from the input block size. Therefore, a two-stage tally-and-allocate strategy is adopted. In the first stage, the sign-bit block is scanned to obtain the run-length statistics and determine the repetition ratio R. Based on these statistics, the appropriate VRLE mode is selected and the required output buffer size is estimated. In the second stage, memory is dynamically allocated according to the estimated encoded size, after which the actual encoding operation is performed. This strategy reduces unnecessary SRAM allocation while ensuring sufficient buffer capacity for the encoded output, thereby improving memory utilization on the resource-constrained sensor node.

### 3.3. Non-Uniform Quantization Coding for the Content-Bit Segment

For the 16-bit content-bit segment, the primary objective is to reduce the data width from 16 bits to 8 bits while limiting the reconstruction error introduced by quantization. The amplitude distribution of mechanical vibration signals is generally non-uniform, and a large proportion of samples may be concentrated in the small-amplitude region under stable operating conditions. Uniform quantization assigns an identical quantization step to the entire amplitude range. Consequently, the same absolute quantization error can produce a relatively larger distortion for small-amplitude components than for large-amplitude components. Non-uniform quantization provides a more suitable alternative by assigning finer quantization resolution to densely populated amplitude regions and coarser resolution to less frequently occurring large-amplitude regions, thereby improving the utilization of the limited output code space [29].

A representative implementation of this principle is the A-Law companding scheme widely used in speech coding [30]. A-Law maps linear PCM samples to an 8-bit logarithmically compressed representation using a piecewise amplitude-dependent coding structure. The proposed method is inspired by this piecewise non-uniform quantization principle, but it does not directly implement the standard A-Law companding algorithm. Specifically, the similarity lies in the use of amplitude-dependent intervals and progressively coarser quantization for larger-magnitude samples, thereby allocating more quantization levels to the small-amplitude region. However, the proposed method is specifically designed for 16-bit mechanical vibration ADC data and uses eight explicitly defined amplitude intervals, a 3-bit interval index, and a 4-bit quantization-level index to generate an 8-bit representation. Therefore, the proposed scheme should be regarded as an A-Law-inspired non-uniform quantization method rather than a direct implementation of the standard A-Law codec.

The proposed scheme retains finer quantization resolution in the small-amplitude region and progressively increases the quantization interval for larger amplitudes, enabling the 16-bit content-bit segment to be represented by an 8-bit code with controlled reconstruction distortion.

#### 3.3.1. Encoding Procedure

**Figure 5:**
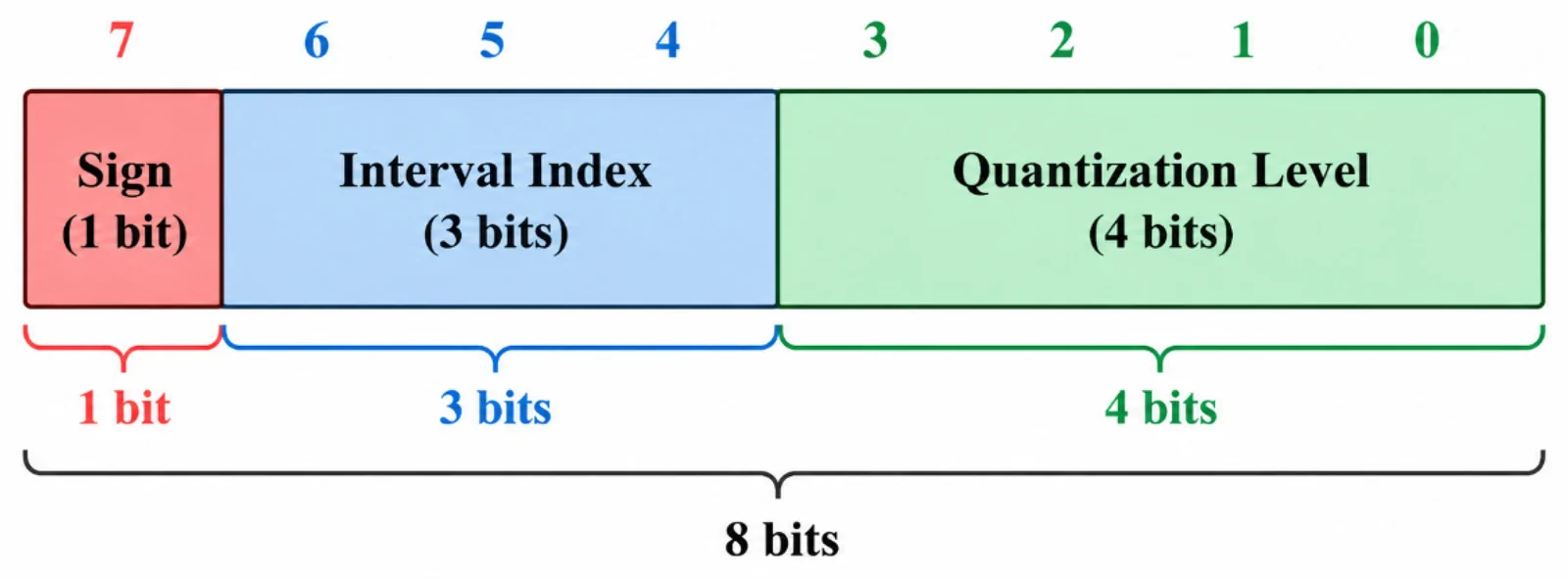
Non-uniform quantization interval partitioning and 8-bit codeword structure.

For the 16-bit content-bit segment (Bits 15–0), the proposed non-uniform quantization scheme maps each input value to an 8-bit codeword consisting of a 1-bit sign field, a 3-bit interval index, and a 4-bit quantization-level index, as illustrated in Figure 5. After the original 24-bit ADC sample is divided into the upper 8-bit sign-bit segment (Bits 23–16) and the lower 16-bit content-bit segment (Bits 15–0), the latter is treated as an independent signed 16-bit value for quantization. Therefore, Bit 15, which is the most significant bit of the extracted 16-bit value, is used to determine its sign. Following the sign convention adopted in A-Law coding, the sign field is defined as:

(4) $$s=\left\{\begin{array}{cc} 1, & b_{15}=0\\ 0, & b_{15}=1 \end{array}\right.$$

where *b*_15_ denotes the most significant bit of the 16-bit content-bit value.

It should be noted that the sign field used in the non-uniform quantization procedure is derived from Bit 15 of the 16-bit content-bit segment, rather than from Bit 23 of the original 24-bit ADC sample. Specifically, following the A-law sign convention, the sign field is set to 1 when Bit 15 is 0 and to 0 when Bit 15 is 1. This operation is performed independently on the lower 16-bit segment, while the upper 8-bit segment, which contains the original 24-bit sign information, is preserved losslessly through VRLE. During reconstruction, the recovered content-bit segment is combined with the losslessly decoded upper 8 bits to form the reconstructed 24-bit sample.

The magnitude range [0, 32768) is then partitioned into eight non-uniform quantization intervals, indexed by *n* = 0, 1, …, 7. Smaller-amplitude values are assigned narrower intervals to provide finer quantization resolution, whereas progressively wider intervals are used for larger amplitudes. Each interval is further divided into 16 quantization levels represented by a 4-bit index.

(5) $$\left\{\begin{array}{ll} 0\leq x<256, & n=0,\\ 128\times 2^{n}\leq x<256\times 2^{n}, & n=1,\ldots ,7. \end{array}\right.$$

Let *x* denote the magnitude of the 16-bit content value to be compressed. The interval index *n* is determined according to (5).

Once the quantization interval *n* is determined, the relative offset *r* of |x| within the interval is computed, and the 4-bit quantization codeword b (0–15) is derived from *r*. The 16-bit content value is thus compressed into an 8-bit codeword: Bit 7 carries the sign, Bits 6–4 carry the 3-bit interval index *n*, and Bits 3–0 carry the 4-bit quantization codeword *b*.

Within each quantization interval, the values are uniformly quantized. Based on the relative offset r, the quantized value b is obtained as:

(6) $$r<\left\{\begin{array}{c} \;\;\;2^{4}\times b,n=0\\ 2^{n+3}\times b,n=1,2,\ldots ,7 \end{array}\right.$$

The 16-bit content value is thus compressed into an 8-bit codeword:

(7) $$n_{8}=s\times 128+n\times 16+b$$

Following the coding convention of A-Law PCM, a fixed bit mask is applied to the 8-bit quantization codeword after the sign, interval, and quantization-level fields are combined. Specifically, Bits 6, 4, 2, and 0 are inverted, while the remaining bits, including the sign bit (Bit 7), are unchanged. This operation is fully reversible and introduces no additional quantization error. During decoding, the same bit mask is applied to recover the original quantization codeword before inverse quantization.

(8) $$\mathrm{codeword}=n_{8}⊕(0101\;\;0101)$$

#### 3.3.2. Decoding and Reconstruction Procedure

The decoding procedure reverses the operations performed during encoding. The received 8-bit quantization codeword *C* is first XORed with the same fixed mask used during encoding:

(9) $$n_{8}=\mathrm{codeword}⊕(0101\;\;0101)$$

Since XOR is self-inverse, this operation restores the original 1 + 3 + 4-bit quantization codeword. The sign field *s*′, interval index *n*′, and quantization-level index *b*′ are then extracted as:

(10) $$s^{\prime }=\left\lfloor \frac{n_{8}}{128}\right\rfloor$$

(11) $$n^{\prime }=\left\lfloor \frac{n_{8}\;\mathrm{mod}\;128}{16}\right\rfloor$$

(12) $$b^{\prime }=n_{8}\;\mathrm{mod}\;16$$

The relative quantized magnitude is calculated from the 4-bit quantization-level index as:

(13) $$\mathrm{QI}=16b^{\prime }$$

The reconstructed magnitude $\hat{x}$ is then obtained according to the decoded interval index:

(14) $$\hat{x}=\left\{\begin{array}{ll} \mathrm{QI}, & n^{\prime }=0,\\ \mathrm{QI}+256, & n^{\prime }=1,\\ (\mathrm{QI}+256)2^{n^{\prime }-1}, & n^{\prime }=2,\ldots ,7. \end{array}\right.$$

Finally, the sign of the reconstructed value is restored according to *s*′, with *s*′ = 1 representing a non-negative value and *s*′ = 0 representing a negative value.

For example, consider a 24-bit ADC sample whose extracted 16-bit content-bit segment is *x* = 100. Its 16-bit two’s-complement representation is 0000 0000 0110 0100, where Bit 15 is 0 and therefore the sign field is set to *s* = 1. Since ∣*x*∣ = 100 < 256, the value belongs to interval *n* = 0, and the quantization index is *b* = ⌊100/16⌋ = 6. The resulting 8-bit codeword before XOR masking is therefore *n*_8_ = (1≪7)∣(0≪4)∣6 = 10000110_2_. After XORing with 0*x*55, the transmitted code is *C* = 11010011_2_. During decoding, *C*⊕0*x*55 recovers *n*_8_, from which *s* = 1, *n* = 0, and *b* = 6 are obtained. The reconstructed value is $\hat{x}=16\times 6=96$, demonstrating the quantization process and the corresponding reconstruction error.

As an additional example, consider a negative 24-bit ADC sample (X = −1000), represented as 0xFFFC18 in 24-bit two’s-complement format. After segmentation, the upper 8-bit segment is 0xFF, while the lower 16-bit content-bit segment is 0xFC18. When interpreted independently as a signed 16-bit value, the latter corresponds to (x = −1000), with the binary representation 1111 1100 0001 1000. Since Bit 15 is 1, the sign field is set to (s = 0). According to (5), the magnitude |x| = 1000 falls within the interval 512 ≤ |*x*| < 1024, corresponding to (n = 2). The relative offset is r = 1000 − 512 = 488, and the quantization step is 2*^n^*^+3^ = 32. Therefore, the quantization index is *b* = ⌊488/32⌋ = 15. The resulting 8-bit codeword before XOR masking is *n*_8_ = (0≪7)∣(2≪4)∣15 = (00101111)_2_. After XORing with 0x55, the transmitted codeword becomes *C* = (01111010)_2_. During decoding, XORing C with 0x55 recovers *n*_8_ = (00101111)_2_, from which *s*′ = 0, *n*′ = 2, and *b*′ = 15 are obtained. According to (13), the relative quantized magnitude is *QI* = 16 × 15 = 240. Using (14), the reconstructed magnitude is (240 + 256) × 2^2−1^ = 992. Since *s*′ = 0, the reconstructed signed 16-bit value is $\hat{x}=-992$, corresponding to the two’s-complement representation 0xFC20. Finally, the reconstructed lower 16-bit segment 0xFC20 is concatenated with the losslessly recovered upper 8-bit segment 0xFF, yielding the reconstructed 24-bit sample $\hat{X}=0\mathrm{xFFFC}20=-992$. The reconstruction error is therefore $X-\hat{X}=-8$. This example demonstrates the correct handling of negative values and the reconstruction of the 24-bit ADC sample using the independently decoded content-bit segment and the losslessly recovered upper 8 bits.

### 3.4. Range Coding

After VRLE and non-uniform quantization, residual statistical redundancy may remain in both the sign-bit and content-bit streams because the encoded symbols generally exhibit non-uniform occurrence probabilities. Range coding is therefore employed as a final entropy-coding stage to further reduce the data size. Range coding is an entropy-coding method closely related to arithmetic coding, in which a symbol sequence is represented by progressively narrowing a numerical interval according to the cumulative probability distribution of the symbols [31].

In the proposed implementation, integer arithmetic is used for interval updates, making Range coding suitable for resource-constrained embedded processing. During encoding, the current interval is subdivided according to the cumulative symbol frequencies, and the subinterval corresponding to the current symbol is selected. As symbols are processed, the interval is repeatedly renormalized to maintain sufficient numerical precision, while stable leading bits are output to the compressed stream. The sign-bit and content-bit streams are processed separately by the Range encoder, and the resulting outputs form the final compressed bitstream.

## 4. Experimental Evaluation

### 4.1. Experimental Platform and Implementation

**Figure 6:**
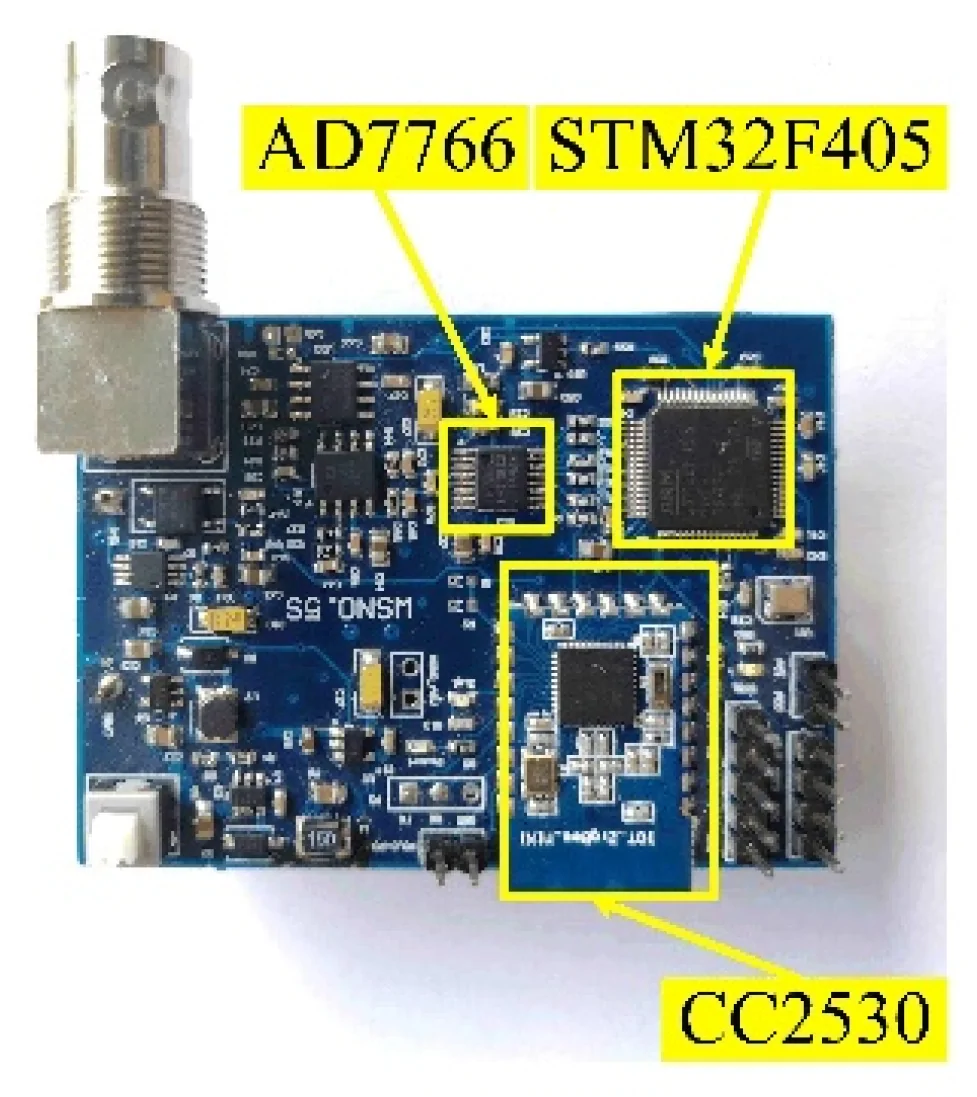
Prototype of the self-developed mechanical vibration WSN sensor node.

To evaluate the proposed compression method under practical operating conditions, the algorithm was implemented on a self-developed mechanical vibration WSN node. The node employs a heterogeneous dual-processor architecture consisting of an STM32F405 microcontroller and a CC2530 wireless SoC. The STM32F405 operates at 168 MHz with 192 KB of SRAM and 1 MB of Flash memory and is responsible for vibration data acquisition, preprocessing, and compression. The CC2530 handles wireless communication and network management based on IEEE 802.15.4. The analog front-end incorporates an independent 24-bit Σ–Δ ADC with a maximum sampling rate of 50 kHz and an SNR of 105 dB. The ADC communicates with the STM32F405 through the SPI interface. A Micro SD card connected through the SDIO interface is used for temporary storage of the acquired vibration data, allowing the STM32F405 to load individual data blocks into SRAM as required. A photograph of the developed sensor node is shown in Figure 6.

The compression algorithm is executed sequentially on the STM32F405. During each processing cycle, a block of 2048 raw ADC samples (6144 bytes) is transferred from the Micro SD card to SRAM via SDIO using DMA. The samples are then separated into an 8-bit sign-bit stream and a 16-bit content-bit stream. The former is compressed using VRLE, whereas the latter is processed using the proposed non-uniform quantization scheme. The two resulting streams are separately processed by Range coding and subsequently assembled into a compressed data packet. The packet is transferred to the CC2530 through the UART interface and transmitted to the data center over the IEEE 802.15.4 wireless link. At the data center, the corresponding decoding operations are performed in reverse order to reconstruct the 24-bit ADC samples. The reconstructed data are then compared with the original measurements to evaluate compression performance and reconstruction accuracy.

### 4.2. Performance Metrics

The performance of the proposed method is evaluated using two metrics: the compression ratio (CR) and the RSNR.

The compression ratio is defined as:

(15) $$\mathrm{CR}=S_{O}/S_{C}$$

where *S_o_* denotes the size of the original data block and *S_c_* denotes the size of the final compressed bitstream generated by the proposed method. Specifically, *S_c_* includes the outputs of the two differentiated compression branches after VRLE and non-uniform quantization, followed by Range coding and the necessary coding information required for decoding. Therefore, *S_c_* represents the total number of bits in the final compressed bitstream rather than the size of a fixed-length intermediate buffer.

The reconstruction signal-to-noise ratio quantifies the overall quality of the reconstructed signal and is calculated as:

(16) $$\mathrm{RSNR}=10\times \log_{10}\frac{x_{\mathrm{rms}}^{2}}{\mathrm{RMS}E^{2}}$$

where $x_{\mathrm{rms}}=\sqrt{\frac{1}{N}\sum_{i}^{n}x_{i}^{2}}$ is the root mean square of the original signal and $\mathrm{RMSE}=\sqrt{\frac{1}{N}\sum_{i}^{n}{\left(x_{i}-{x_{i}}^{\prime }\right)}^{2}}$ is the root mean square error between the original and reconstructed signals. Since the 24-bit ADC data and the converted vibration data differ only by a constant scaling factor, the RSNR can be directly computed from the raw ADC sample values.

To evaluate the applicability of the proposed method to mechanical dynamic signals with different temporal and spectral characteristics, compression experiments were conducted on two representative types of data. The first type consisted of periodic vibration signals acquired from a rotor test bench under steady operating conditions. The second type comprised aperiodic impact-force and structural-response signals obtained from hammer-impact modal testing of a steel frame. Compared with the periodic rotor vibration signals, the modal-test signals exhibit substantially different temporal characteristics and spectral energy distributions. These two types of data therefore provide complementary test cases for evaluating the compression performance and reconstruction accuracy of the proposed method under different signal characteristics.

### 4.3. Sensitivity Analysis of VRLE Thresholds

To evaluate the influence of the VRLE thresholds on compression performance, a sensitivity analysis was conducted using 20 rotor vibration datasets. The upper threshold *R_H_* was varied from 0.4 to 0.8 in increments of 0.1 while keeping (*R_L_* = 0.3). Similarly, the lower threshold *R_L_* was varied from 0.1 to 0.5 in increments of 0.1 while keeping (*R_H_* = 0.6). All other compression parameters, including the block size of 2048 samples, were kept unchanged. The mean CR and standard deviation were calculated across the datasets for each threshold configuration.

**Figure 7:**
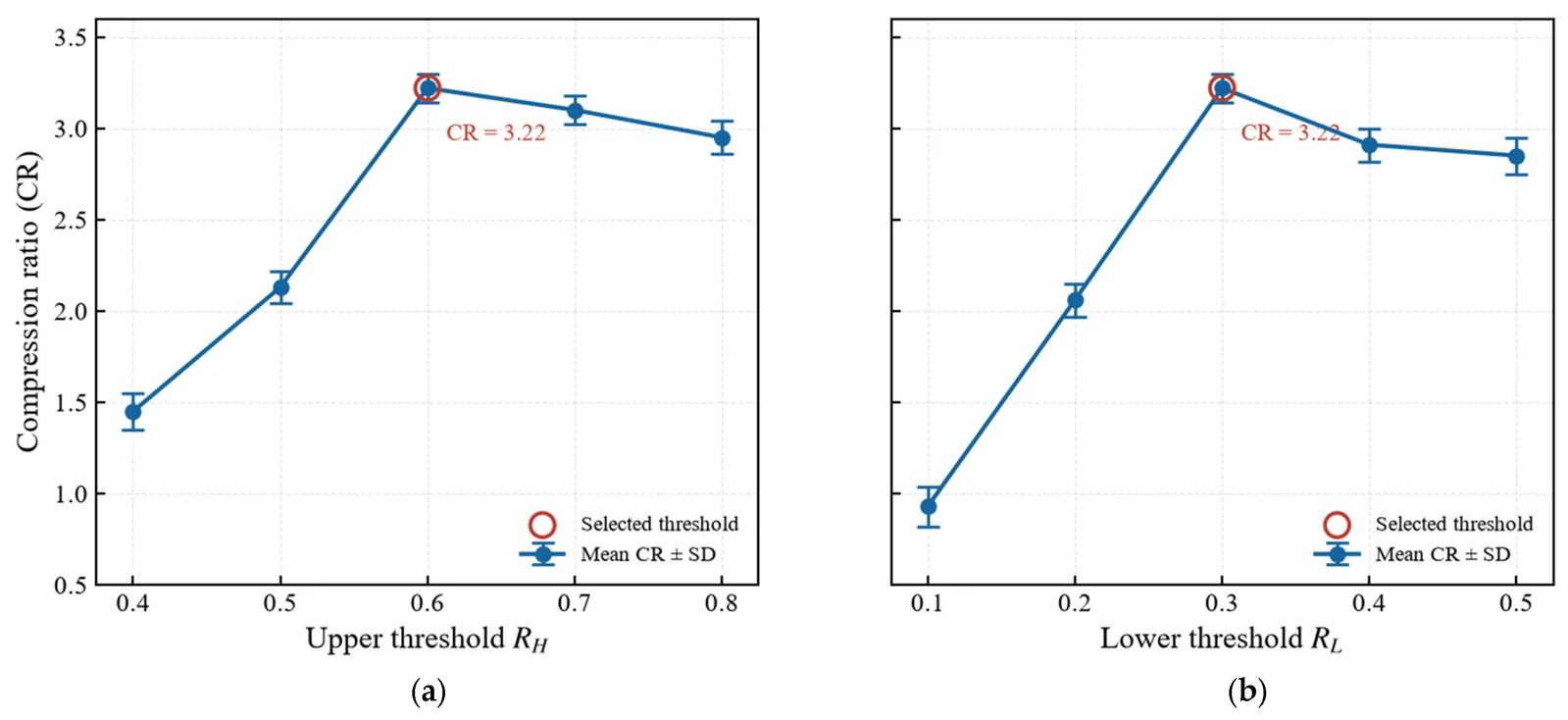
Sensitivity analysis of VRLE thresholds based on the mean compression ratio and standard deviation across 20 rotor vibration datasets: (**a**) influence of *R_H_* with *R_L_* = 0.3; (**b**) influence of *R_L_* with *R_H_* = 0.6. Error bars indicate ±1 standard deviation.

As shown in Figure 7a, the mean CR increases from 1.45 to 3.22 as *R_H_* increases from 0.4 to 0.6, and subsequently decreases to 2.95 at (*R_H_* = 0.8). Similarly, Figure 7b shows that the mean CR increases from 0.93 to 3.22 as (*R_L_*) increases from 0.1 to 0.3, before decreasing to 2.85 at (R*_L_* = 0.5). These variations reflect the influence of threshold selection on the switching among full run-length, mixed, and raw pass-through modes. Lower threshold values may cause blocks with insufficient temporal redundancy to enter more expensive encoding modes, whereas excessively high thresholds may prevent the encoder from exploiting otherwise compressible repeated sequences.

Among the evaluated configurations, the combination (*R_H_* = 0.6) and (*R_L_* = 0.3) achieves the highest mean CR of 3.22. Therefore, these thresholds were selected for the proposed VRLE scheme.

### 4.4. Compression Performance for Periodic Vibration Signals

**Figure 8:**
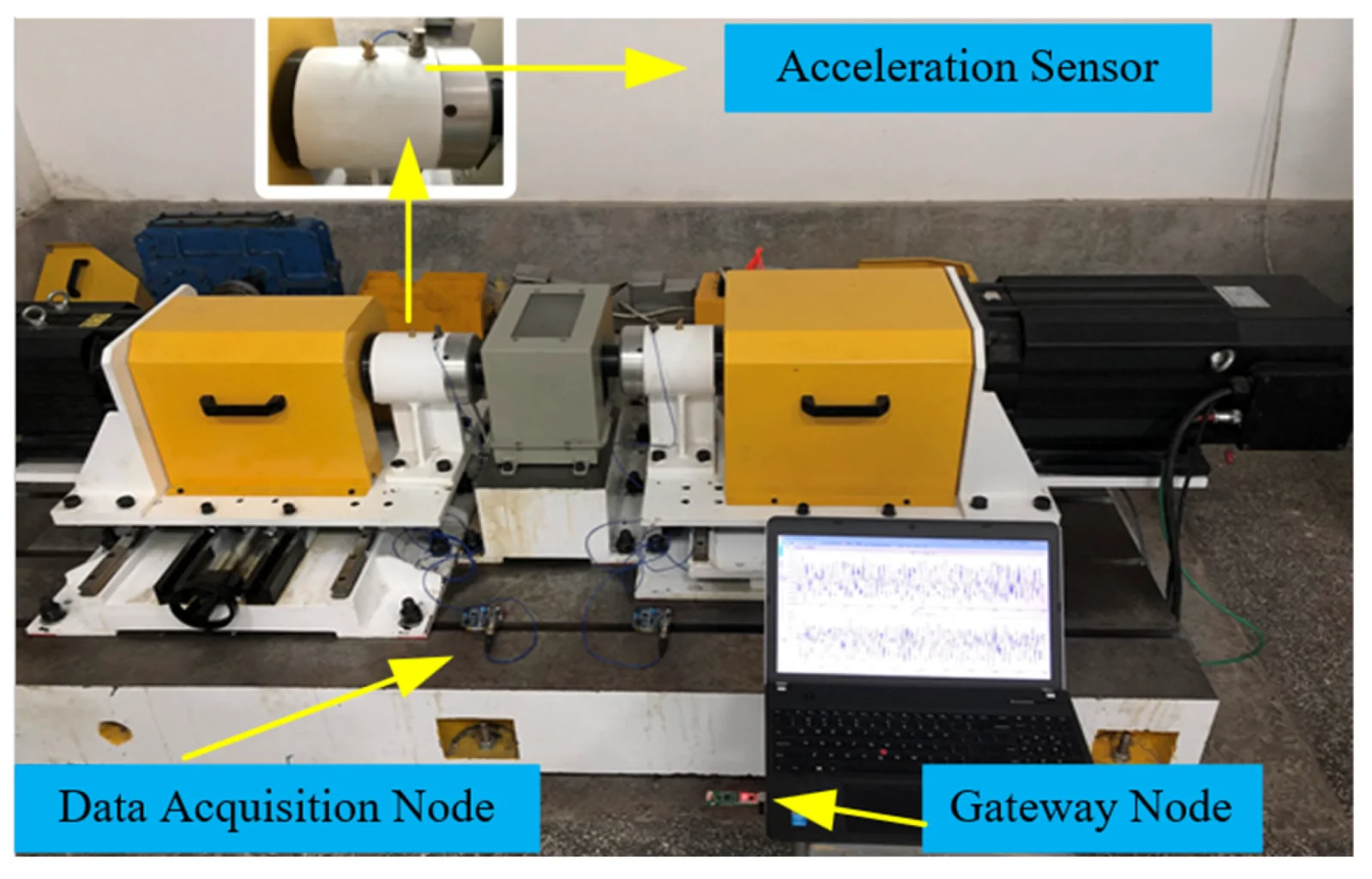
Experimental setup for periodic rotor vibration measurements.

The periodic vibration experiment was conducted on a rotor test bench, as shown in Figure 8. The test bench consists of a variable-frequency drive motor, a flexible coupling, two rolling-bearing housings, and a rotor disk. The WSN sensor node was magnetically mounted on the input-shaft bearing housing to measure radial vibration acceleration in the vertical direction. The sampling frequency was set to 25.6 kHz with a 24-bit ADC resolution, and each measurement lasted 5 s. Consequently, each acquisition contained 128,000 samples, corresponding to 384,000 bytes (375 KiB) of raw ADC data.

During the experiment, the acquired raw data were first stored on the Micro SD card. After the 5-s acquisition was completed, the data center issued a transmission command through the wireless link. The sensor node then read the stored data in blocks of 2048 samples, compressed each block using the proposed method, and transmitted the resulting compressed packets to the data center. The received data were decompressed and reconstructed at the data center, and the reconstructed signals were compared with the corresponding original data to evaluate the compression performance and reconstruction accuracy.

**Figure 9:**
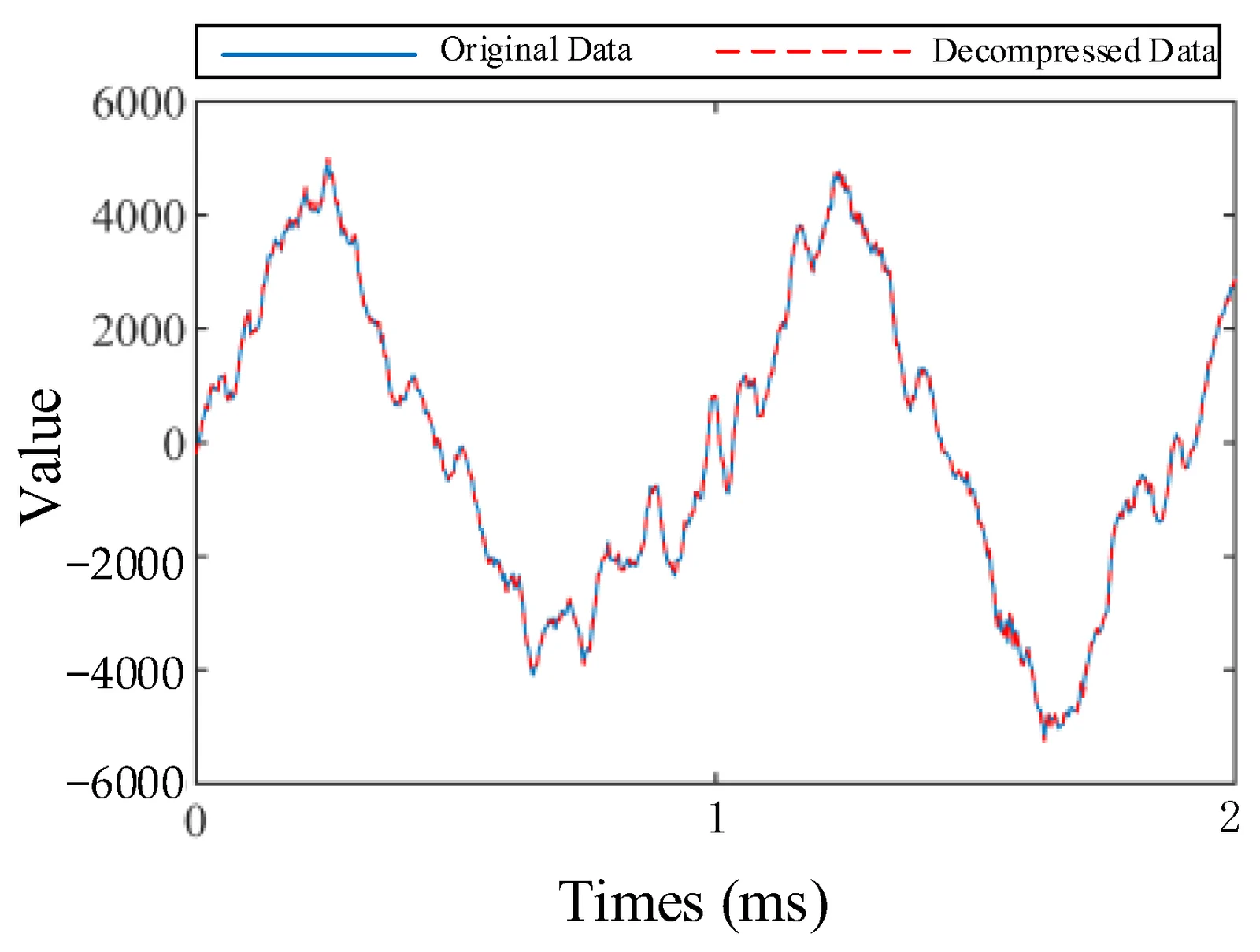
Comparison of original and decompressed data for periodic signals.

A total of 20 independent datasets were acquired and compressed in this experiment. The proposed method achieved a mean CR of 3.22 with a standard deviation of 0.08, while the mean RSNR was 37.4 dB with a standard deviation of 0.6 dB. Figure 9 compares the original and reconstructed time-domain waveforms from a representative test. The two waveforms exhibit close agreement in both the overall waveform profile and local variations, with no visually significant differences. The reconstruction error is approximately within ±30 ADC units, corresponding to less than 0.0004% of the 24-bit positive full-scale magnitude (8,388,608). These results indicate that the proposed method achieves a compression ratio greater than 3 while maintaining high waveform reconstruction accuracy for periodic rotor vibration signals.

To further illustrate the contributions of the individual compression stages, a representative block containing 2048 samples from the original vibration dataset was analyzed. The original 24-bit data occupied 6144 bytes, comprising 2048 bytes in the sign-bit segment and 4096 bytes in the content-bit segment. For this block, a simplified run-length representation reduced the sign-bit segment to approximately 90 bytes, corresponding to a reduction of 1958 bytes (31.87% of the original data size), while non-uniform quantization reduced the content-bit segment from 4096 to 2048 bytes, contributing a further reduction of 2048 bytes (33.33%). Consequently, the combined data size before Range coding was approximately 2138 bytes. After Range coding, the final compressed data size was approximately 1903 bytes, indicating an additional reduction of 235 bytes (3.82%). Overall, the data size was reduced by 69.03%, corresponding to a compression ratio of approximately 3.23.

### 4.5. Aperiodic Modal-Testing Signal Experiment

**Figure 10:**
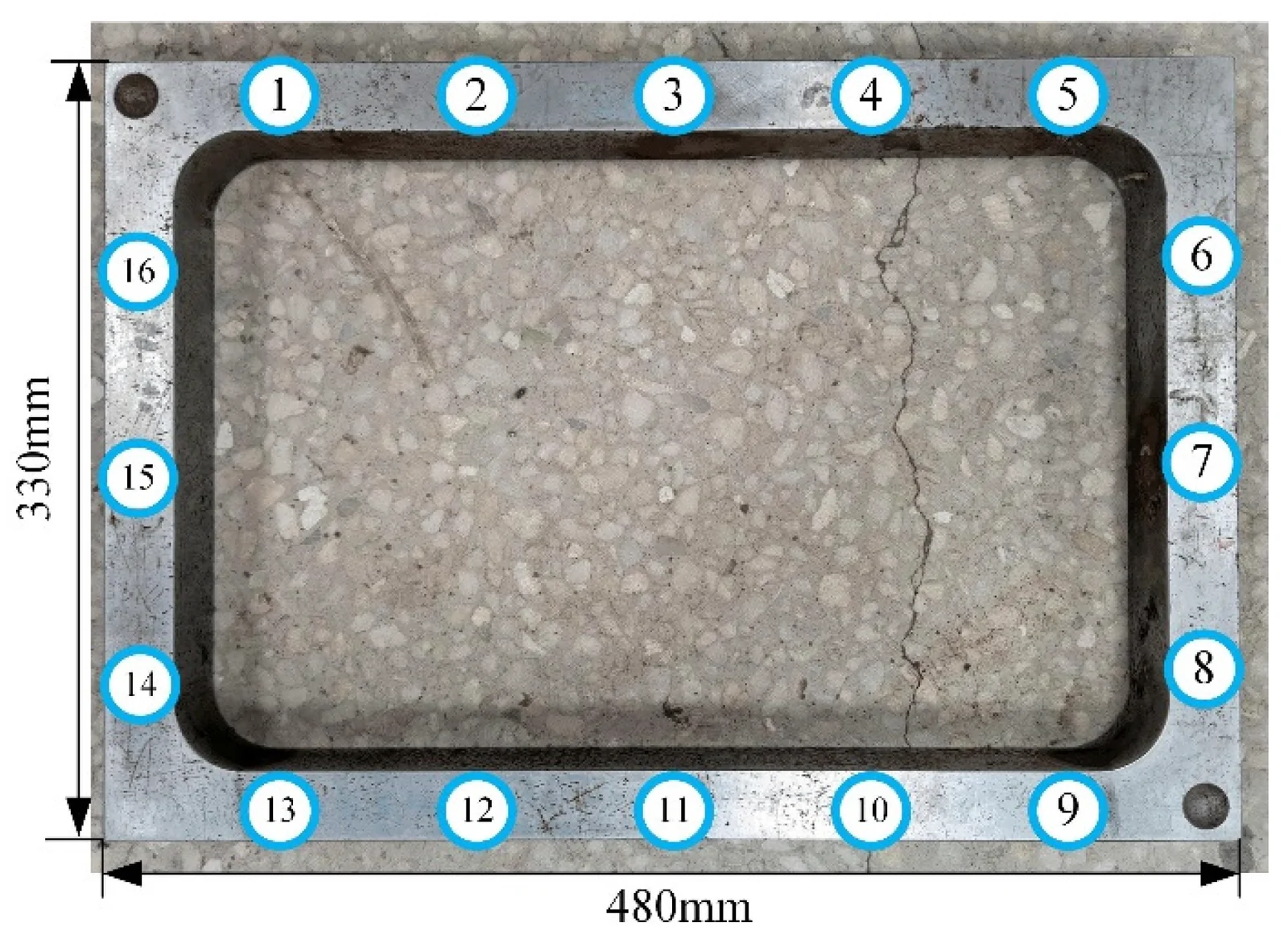
Division of measurement points on the steel frame.

The aperiodic compression experiment was conducted on a rectangular steel frame using hammer-impact modal testing. The experiment was designed to evaluate whether the proposed compression method can preserve structural modal characteristics, including natural frequencies and damping ratios. The frame has overall dimensions of 480 mm × 330 mm × 45 mm and a rectangular cross-section of 30 mm × 45 mm and is made of Q235 structural steel. To characterize the global vibration modes of the frame in the vertical bending direction, 16 measurement points were uniformly distributed over the frame in a 4 × 4 grid, as shown in Figure 10.

**Figure 11:**
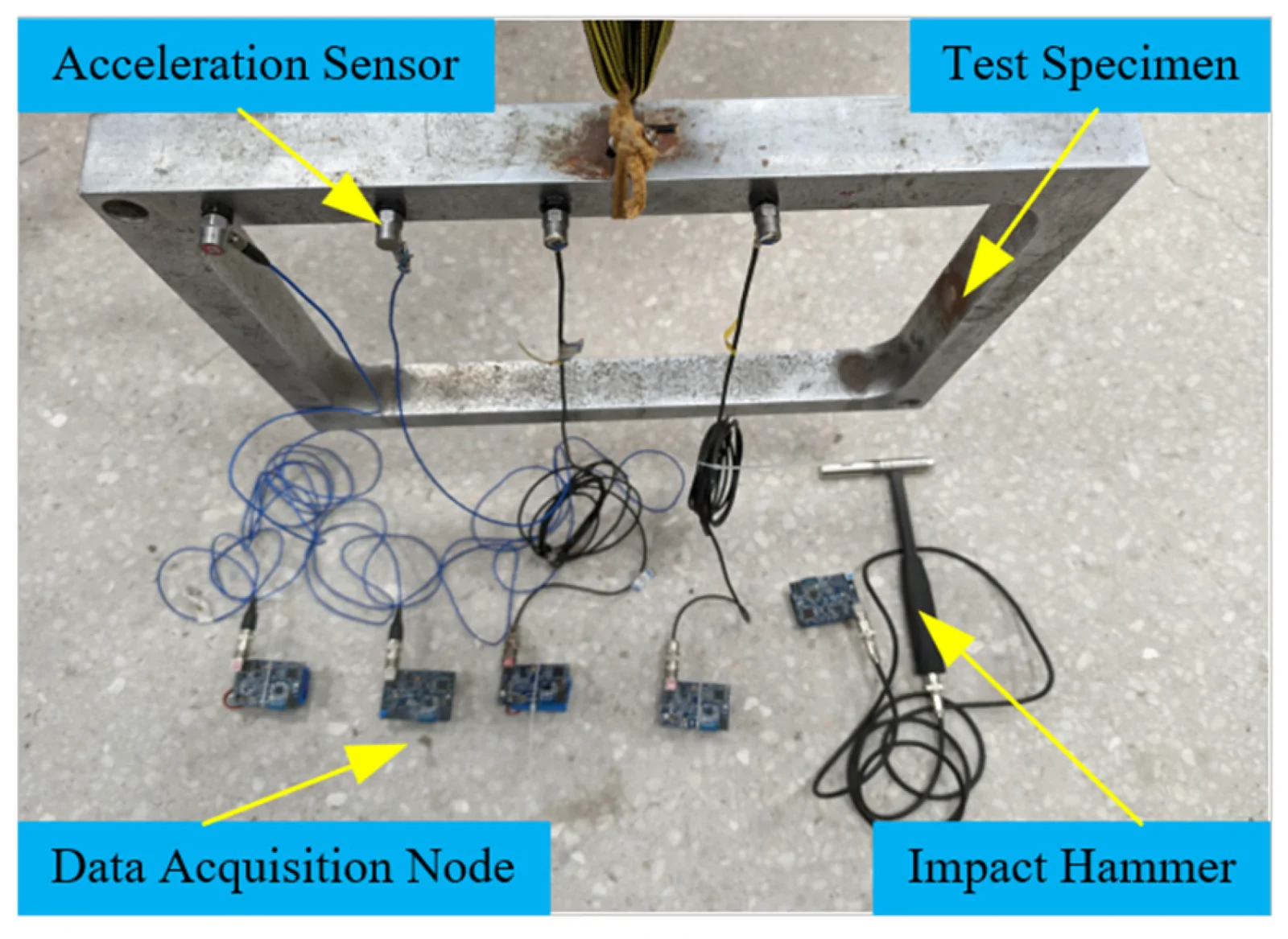
Schematic diagram of the modal experiment setup.

The modal testing system consisted of five self-developed WSN acquisition nodes and one gateway node, as shown in Figure 11. One acquisition node was used to trigger the measurement and acquire the impact-force signal from an instrumented hammer (PCB 086C03, sensitivity: 2.25 mV/N). The other four nodes were used to acquire structural acceleration responses, each equipped with a uniaxial IEPE accelerometer with a sensitivity of 100 mV/g. To cover all 16 measurement points, the four response nodes were sequentially relocated to different positions on the frame. For each measurement configuration, five independent hammer impacts were performed to reduce the influence of variations in the impact location and force amplitude.

All acquisition channels were sampled at 12.5 kHz, with 50,000 samples recorded per channel for each impact. Each dataset therefore contained 150,016 bytes, including 150,000 bytes of 24-bit measurement data and a 16-byte header storing the node ID, timestamp, and acquisition parameters. After completion of the modal test, both the original data and the corresponding compressed–reconstructed data were independently processed using the self-developed SP-Model 3.0 modal analysis software. Modal parameters were identified using the PolyMAX method, and the results obtained from the two datasets were compared to evaluate the influence of compression on the identified structural modal characteristics.

Taking the impact-force signal and the acceleration response at measurement point 1 as representative examples, the proposed method achieved a CR of 3.16 and an RSNR of 46.49 dB for the impact-force signal, and a CR of 2.66 and an RSNR of 54.2 dB for the acceleration response. The difference in RSNR reflects the distinct temporal and amplitude characteristics of the two signals. The impact-force signal is characterized by a sharp transient peak followed by rapid decay, whereas the structural response exhibits a smoother decaying waveform dominated by the dynamic response of the frame. Consequently, the two signals exhibit different reconstruction errors under the same non-uniform quantization scheme, with the acceleration response achieving the higher RSNR in this experiment.

**Figure 12:**
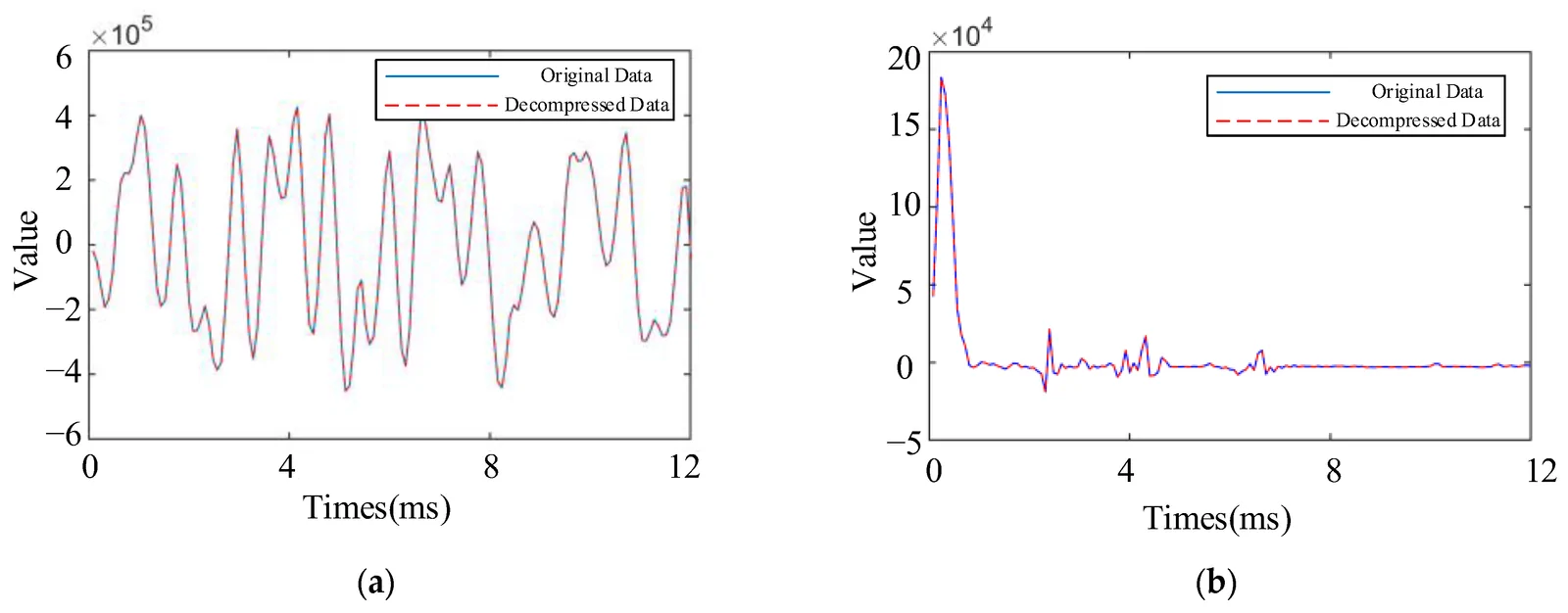
Comparison of original and decompressed data for aperiodic signals. (**a**) Force hammer excitation signal; (**b**) Vibration response signal at measurement point 1.

Figure 12a,b compare the original and reconstructed time-domain waveforms of the impact-force and acceleration-response signals, respectively. In both cases, the reconstructed waveforms closely follow the original signals in terms of the overall waveform profile, peak location, and decay behavior. These results demonstrate that the proposed method can effectively compress aperiodic transient signals while preserving their principal time-domain characteristics.

**Figure 13:**
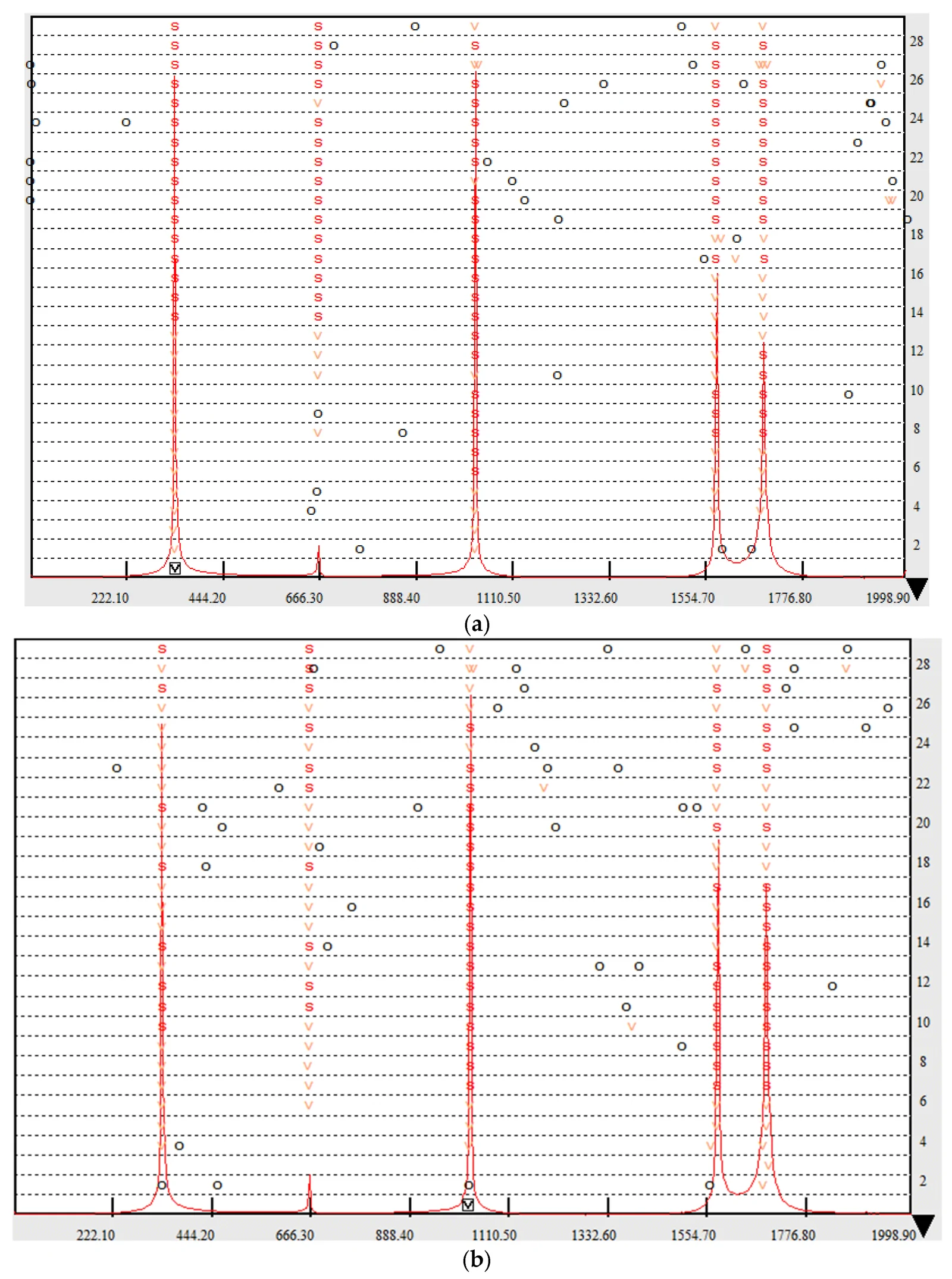
Comparison of stability diagrams between original and decompressed data. (**a**) Stability diagram obtained from the original data; (**b**) stability diagram obtained from the decompressed data. Symbols: s—stable pole; v—stable frequency and mode shape vector; o—stable frequency.

Figure 13 presents the stabilization diagrams obtained from the original and compressed–reconstructed data using SP-Model 3.0. In each diagram, the symbol “s” denotes a stable pole that simultaneously satisfies the stability criteria for natural frequency and damping ratio. The horizontal axis represents frequency (Hz), and the vertical axis represents model order. As shown in Figure 13a,b, the stable poles identified from the compressed–reconstructed data exhibit a distribution similar to that obtained from the original data. No substantial additional pole clusters or obvious shifts in the dominant stable poles are observed after compression and reconstruction. This indicates that the proposed compression method has only a limited influence on the stabilization characteristics used for modal identification.

Table 3 further compares the natural frequencies and damping ratios of the first five modes identified from the original and compressed–reconstructed data. The maximum relative error in natural frequency is 0.03%, occurring for the third and fifth modes. The damping ratios obtained from the two datasets also show close agreement, with only limited deviations after compression and reconstruction. These results demonstrate that the proposed method preserves the principal modal information quantitatively evaluated in this study, particularly the natural frequencies and damping ratios.

### 4.6. Comparison with an Existing Vibration Compression Method

To further evaluate the effectiveness of the proposed method, a direct comparison was conducted with the hybrid DCT-modal compression method proposed by Pinto et al. [32]. This method was selected as a representative vibration-signal compression approach that combines transform-domain coding with modal-information-based signal prediction. For the baseline implementation, the vibration signals were processed using a DCT block length of 2048 samples. Following the coefficient-thresholding strategy described in [32], the DCT threshold was defined as $T_{h}=\alpha \;\underset{k}{\max}\left|C_{k}\right|$, where *C_k_* denotes the DCT coefficients and *α* is the threshold factor. In this study, *α* was set to 0.010, and coefficients with absolute values below *T_h_* were set to zero. The resulting coefficients and prediction residuals were processed using 10-bit linear scalar quantization and Golomb entropy coding, with the Golomb parameter *m* set to 1. The threshold factor was selected to obtain a compression ratio comparable to that of the proposed method, while the other coding parameters were kept fixed. Both methods were evaluated using the same rotor vibration dataset, and RSNR was calculated using the same definition to ensure consistency in the evaluation of reconstruction quality.

As shown in Table 4 the hybrid DCT-modal method achieves a compression ratio of approximately 3.22 with an RSNR of 32.7 dB, whereas the proposed method achieves a CR of 3.22 and an RSNR of 37.4 dB. Thus, at a comparable compression ratio, the proposed method improves the RSNR by approximately 4.7 dB compared with the hybrid DCT-modal method. This result indicates that the proposed method provides better reconstruction fidelity at a similar compression level for the investigated mechanical vibration data.

### 4.7. Resource and Energy Consumption Evaluation

To further evaluate the practical feasibility of the proposed compression method on resource-constrained WSN nodes, its computational complexity was theoretically analyzed, while the processing time and energy consumption were experimentally measured on the STM32F405-based sensor node, and the relevant energy calculation methods refer to Ref. [33].

The computational complexity was analyzed according to the main processing stages. For a data block containing N raw ADC samples, data segmentation requires a fixed number of bitwise operations per sample, resulting in a time complexity of O(N). The VRLE stage sequentially scans the sign-bit segment to identify repeated sequences and select the appropriate encoding mode, leading to a complexity of O(N). Similarly, the non-uniform quantization stage processes each content-bit sample through a fixed number of interval comparisons and quantization operations, resulting in O(N) complexity. The subsequent Range coding stage sequentially encodes the generated symbols. Assuming a bounded number of operations per symbol, its computational complexity is also O(N). Therefore, the overall time complexity of the proposed compression method is O(N), indicating that the computational cost scales linearly with the number of input samples.

To assess the actual computational performance, experiments were conducted on the STM32F405-based sensor node. The evaluation was performed on a block basis, with each block containing 2048 raw 24-bit ADC samples, corresponding to 6 KiB of input data. The processing time was measured from the beginning of compression processing to the generation of the final compressed bitstream, including VRLE, non-uniform quantization, and Range coding. The energy consumption was determined from the measured supply voltage and current during the compression procedure. Wireless data transmission was excluded from both measurements.

As shown in Table 5, the compression procedure requires 466 ms and consumes 137.9 mJ per block, corresponding to an average processing throughput of approximately 4.40 kSamples/s. At an average compression ratio of 3.22, the original 6 KiB data block is reduced to approximately 1.86 KiB, corresponding to a reduction of approximately 68.9% in the data payload to be transmitted. This reduction can shorten the wireless payload transmission time and potentially reduce communication energy consumption under identical transmission conditions. The estimated peak SRAM usage was approximately 26.93 KiB, accounting for 14.03% of the 192 KiB SRAM available on the STM32F405 microcontroller.

It should be noted that the developed WSN employs a store-and-forward architecture, in which the acquired vibration data are first stored on a Micro SD card and subsequently compressed upon receiving a transmission command. Therefore, compression is performed independently of the data acquisition process and does not interfere with continuous vibration data collection. Although the compression processing time contributes to the overall data retrieval latency, the proposed method can be implemented on the STM32F405-based sensor node without requiring real-time compression during signal acquisition.

## 5. Conclusions

This paper proposed a two-stage compression method based on byte-level data segmentation and non-uniform quantization for high-resolution mechanical vibration data in resource-constrained WSNs. By exploiting the distinct statistical characteristics of different byte segments in 24-bit raw ADC data, each sample is divided into an 8-bit sign-bit segment and a 16-bit content-bit segment. The sign-bit segment is losslessly compressed using adaptive VRLE, while the content-bit segment is reduced through an eight-interval non-uniform quantization scheme inspired by the A-Law companding scheme. Range coding is subsequently applied to further reduce residual statistical redundancy. This differentiated compression strategy enables efficient data reduction while limiting the reconstruction distortion introduced by quantization.

The proposed method was implemented on a self-developed mechanical vibration WSN platform and evaluated using periodic rotor vibration signals and aperiodic signals acquired from hammer-impact modal testing. For the periodic rotor vibration data, the method achieved a mean CR of 3.22 and a mean RSNR of 37.4 dB. For the modal-testing data, CR values of 3.16 and 2.66 were obtained for the impact-force and structural-response signals, respectively, with corresponding RSNR values of 46.49 and 54.2 dB. Modal analysis further showed that the maximum relative error in the first five identified natural frequencies between the original and compressed–reconstructed data was only 0.03%. These results demonstrate that the proposed method can substantially reduce the volume of high-resolution vibration data while maintaining high reconstruction accuracy and preserving the principal modal information required for subsequent structural dynamic analysis.

Future work will focus on improving the adaptability and system-level performance of the proposed method. Online estimation of signal amplitude distributions will be investigated to adaptively adjust the non-uniform quantization intervals under varying operating conditions. The byte-level segmentation strategy may also be combined with other data-reduction techniques to further improve compression efficiency. In addition, experiments in multi-hop WSNs will be conducted to evaluate energy consumption, transmission latency, and communication reliability under different network and channel conditions.

## Figures and Tables

**Table 1:** Sample points and their binary two’s complement representations.

Sampling Point	Sampling Values	24-Bit Complement Code		
		Bits 23–16	Bits 15–8	Bits 7–0
a	2037	0000,0000	0000,0111	1111,0101
b	2330	0000,0000	0000,1001	0001,1010
c	2612	0000,0000	0000,1010	0011,0100
d	2748	0000,0000	0000,1010	1011,1100
e	3042	0000,0000	0000,1011	1110,0010
f	3168	0000,0000	0000,1100	0110,0000
g	3256	0000,0000	0000,1100	1011,1000

**Table 2:** Byte-level statistical characteristics of experimental 24-bit ADC vibration data.

Byte Segment	Distinct Values	Repetition Ratio (%)	Average Run Length
Upper byte (Bits 23–16)	2	98.09	52.44
Middle byte (Bits 15–8)	62	52.24	2.09
Lower byte (Bits 7–0)	256	0.40	1.00

**Table 3:** Comparison of modal parameters between the original data and the decompressed reconstructed data for the first five modes.

Rank	The Original Data		The Compressed–Reconstructed Data		Relative Frequency Error (%)
	Characteristic Frequency (Hz)	Damp Ratio (%)	Characteristic Frequency (Hz)	Damp Ratio (%)	
1	330.191	0.010	330.186	0.008	0.01
2	658.120	0.076	658.216	0.090	0.01
3	1017.030	0.038	1017.409	0.039	0.03
4	1566.485	0.026	1566.734	0.023	0.01
5	1676.270	0.071	1676.774	0.072	0.03

**Table 4:** Comparison between the proposed method and the hybrid DCT-modal compression method.

Method	CR	RSNR (dB)
Hybrid DCT-modal [32]	≈3.22	32.7
Proposed method	3.22	37.4

**Table 5:** Computational complexity, processing performance, and energy consumption of the proposed method.

Metric	Result
Block size	2048 samples
Raw data size	6 KiB
Processing time	466 ms
Theoretical time complexity	*O*(*N*)
Peak memory usage	26.93 KiB
Energy per block	137.9 mJ

## Data Availability

The original contributions presented in this study are included in the article. Further inquiries can be directed to the corresponding author.

## Abbreviations

The following abbreviations are used in this manuscript:

WSNs	wireless sensor networks
ADC	analog-to-digital converter
VRLE	variable run-length encoding
RSNR	reconstruction signal-to-noise
CS	compressed sensing
DCT	discrete cosine transform
CWRU	case western reserve university
RLE	run-length encoding
Zero-RLE	zero-run-length encoding
CR	compression ratio
